# Social Media and Health Care (Part II): Narrative Review of Social Media Use by Patients

**DOI:** 10.2196/30379

**Published:** 2022-01-07

**Authors:** Deema Farsi, Hector R Martinez-Menchaca, Mohammad Ahmed, Nada Farsi

**Affiliations:** 1 Department of Pediatric Dentistry Faculty of Dentistry King Abdulaziz University Jeddah Saudi Arabia; 2 Department of Comprehensive Dentistry School of Dentistry University of Louisville Louisville, KY United States; 3 Dubai Health Authority Dubai United Arab Emirates; 4 Department of Dental Public Health Faculty of Dentistry King Abdulaziz University Jeddah Saudi Arabia

**Keywords:** social media, social networking, internet, health care, COVID-19, patient, telemedicine, mobile phone

## Abstract

**Background:**

People are now connected in a borderless web-based world. The modern public, especially the younger generation, relies heavily on the internet as the main source of health-related information. In health care, patients can use social media for more tailored uses such as telemedicine, finding a provider, and for peer support.

**Objective:**

The aim of this narrative review is to discuss how social media has been used in the health care industry from the perspective of patients and describe the main issues surrounding its use in health care.

**Methods:**

Between March and June 2020, a review of the literature was conducted on PubMed, Google Scholar, and Web of Science for English studies that were published since 2007 and discussed the use of social media in health care. In addition to only English publications that discussed the use of social media by patients, publications pertaining to ethical and legal considerations in the use of social media were included. The studies were then categorized as *health information*, *telemedicine*, *finding a health care provider*, *peer support and sharing experiences*, and *influencing positive health behavior*. In addition, two more sections were added to the review: *issues pertaining to social media use in health care* and *ethical considerations*.

**Results:**

Initially, 75 studies were included. As the study proceeded, more studies were included, and a total of 91 studies were reviewed, complemented by 1 textbook chapter and 13 web references. Approximately half of the studies were reviews. The first study was published in 2009, and the last was published in 2021, with more than half of the studies published in the last 5 years. The studies were mostly from the United States (n=40), followed by Europe (n=13), and the least from India (n=1). WhatsApp or WeChat was the most investigated social media platform.

**Conclusions:**

Social media can be used by the public and patients to improve their health and knowledge. However, due diligence must be practiced to assess the credibility of the information obtained and its source. Health care providers, patients, and the public need not forget the risks associated with the use of social media. The limitations and shortcomings of the use of social media by patients should be understood.

## Introduction

### Background

There has been an inexorable increase in digitization over the last 2 decades. Over the years, internet use has remarkably developed, in a way that its use has become effortlessly easy. Websites have been developed into user-friendly apps, mobile phones have become smartphones, and internet coverage has become broader than ever. Interactive websites (Web 2.0) are increasingly overshadowing traditional static websites. Web 2.0 is a term that refers to different types of websites and applications that allow any user to generate content and share it on the web in a web-based community. Social media is a type of Web 2.0 that has been recently introduced as internet-based websites and apps, where user-generated content is created and conveniently exchanged with other users [1]. It is designed as a space for people to obtain information, share experiences, build communities, connect electronically both informally and professionally, and link them to others with common interests, which led to the emergence of the term *self-media*. Users generally need to create a profile or account on the vector and then determine with whom to share it, whether it is a list of known users with similar interests or a broader public community that has access to the vector.

### Research in Context

As the consumption of social media has grown, it has become an essential tool used in many industries. In health care, traditional services have been complemented by social media. A simple search on PubMed with the words *social media* would yield several studies, reflecting how relevant the topic is to health care. Although the vast majority of studies investigated social media from the perspective of a health care provider (HCP), there is an abundance of studies that investigated how patients and the public are using it as a resource to supplement traditional health care. Studies varied in their aims, designs, and methodology, and presented mixed findings. Although most studies found promising results, some findings highlighted several limitations and negative issues regarding the use of social media by patients [2-8]. Most included reviews have focused on 1 or 2 main domains of the use of social media in health care such as telemedicine and smoking cessation [9,10]. To our knowledge, no review has holistically discussed the use of social media from the perspective of a patient. In this narrative review, we try to answer the question, “In what ways have patients used social media in relation to health care?” by accumulating, summarizing, and reorganizing findings from published literature.

### Objectives

This review aims to discuss how social media has been an essential tool in the health care industry from the perspective of patients. The discussion is supplemented with a discussion on issues pertaining to the use of social media and the ethical considerations that emerged from the literature.

## Methods

### Methodology Overview

This review is a continuation of the findings presented in *Social Media and Healthcare, Part 1: Literature Review of Social Media Use by Health Care Providers*, which discussed the use of social media in the health care industry from the perspective of an HCP [11]. The original plan was to conduct a general review on the use of social media in health care. Owing to the abundance of information, a decision was made to divide the findings into 2 reviews.

### Search Strategy and Information Sources

In the first phase, a comprehensive search on PubMed, Google Scholar, and Web of Science was conducted in March and April 2020 for medical publications on the use of social media in health care in English from 2007 to date. A combination of the following keywords was used to search for relevant articles: *social media* (Medical Subject Headings [MeSH] term) OR *social networking/social network* OR *internet* (MeSH term) OR *Instagram* OR *Facebook* OR *WhatsApp* OR *LinkedIn* OR *YouTube* OR *Twitter* AND *health care* OR *health* (MeSH term) OR *medicine* (MeSH term) OR *physician* (MeSH term) OR *nursing* (subheading) OR *dentistry* (MeSH term) OR *telemedicine* (MeSH term), *recruitment*, OR *education* (subheading) OR *career* OR *behavior/behaviour* (MeSH term) OR *research* (MeSH term). As studies emerged, a second search was conducted in June 2020 with the following combinations: *social media* (MeSH term) OR *social networking* OR *internet* (MeSH term) AND *legal liability* (MeSH term) OR *professionalism* (MeSH term) OR *impact* (MeSH term) OR *ethics* (MeSH term) OR *limitation* OR *harm*.

### Screening Process

An EndNote (EndNote 20; Clarivate Analytics) library was created, in which the articles were entered and duplicate publications were removed. For articles to be included, they had to (1) be about social media and health care from the perspective of patients; (2) be in the English language; (3) have accessible full text; and (4) be published in 2007 or later. Exclusion criteria were as follows: (1) abstracts only, without full text; (2) non-English; and (3) irrelevant, such as those discussing social media use from the perspective of an HCP or the use of non-Web 2.0 applications. Reviews and observational and experimental studies were included, with no exclusion based on the study design. The eligibility of the titles and abstracts was also assessed. Finally, the full texts were retrieved. Manual reference screening of the included studies was performed to locate other relevant articles.

### Categorization

On the basis of the key outcomes, articles were initially divided into two groups: *patient/the public* and *other relevant issues*. As more information was obtained, the latter was further divided into two groups: *issues pertaining to social media use in health care* and *ethical considerations*. *Issues pertaining to social media use in health care* covered studies on the limitations, negative effects, and harms of use of social media in health care that emerged from the literature. *Ethical considerations* presented information about legal and ethical issues pertaining to the use of social media in health care.

To best present the findings, the group titled *patient/the public* was subsequently divided into 4 subgroups. The first subgroup was *health information*; although this point was discussed in the first review, in this part we have discussed how patients receive information, rather than how HCPs disseminate it. The second subgroup was *telemedicine*; issues pertaining to the use of telemedicine by patients were discussed. *Finding an HCP* was the mirror image of the group named *career development/practice promotion,* which was discussed in the first review. In the previous review, we discussed how HCPs use social media to market themselves and their practice, whereas in this study, we explored the impact of this on patients’ decision-making. The fourth subgroup was *peer support and sharing experiences*, which was unique to patients and the public, and discussed how social media is used among patients for compassion and as a digital word of mouth.

In the first review, a section titled *influencing positive health behavior* was comprehensive. After reviewing it, a decision was made to move it to this review as a fifth group, as it was more relevant to patients than HCPs.

## Results

### Overview

In this section, the search results in terms of the included publications are presented. The findings pertaining to the content of the individual studies were categorized and are presented in the *Discussion* section.

### Search Results

**Figure 1:**
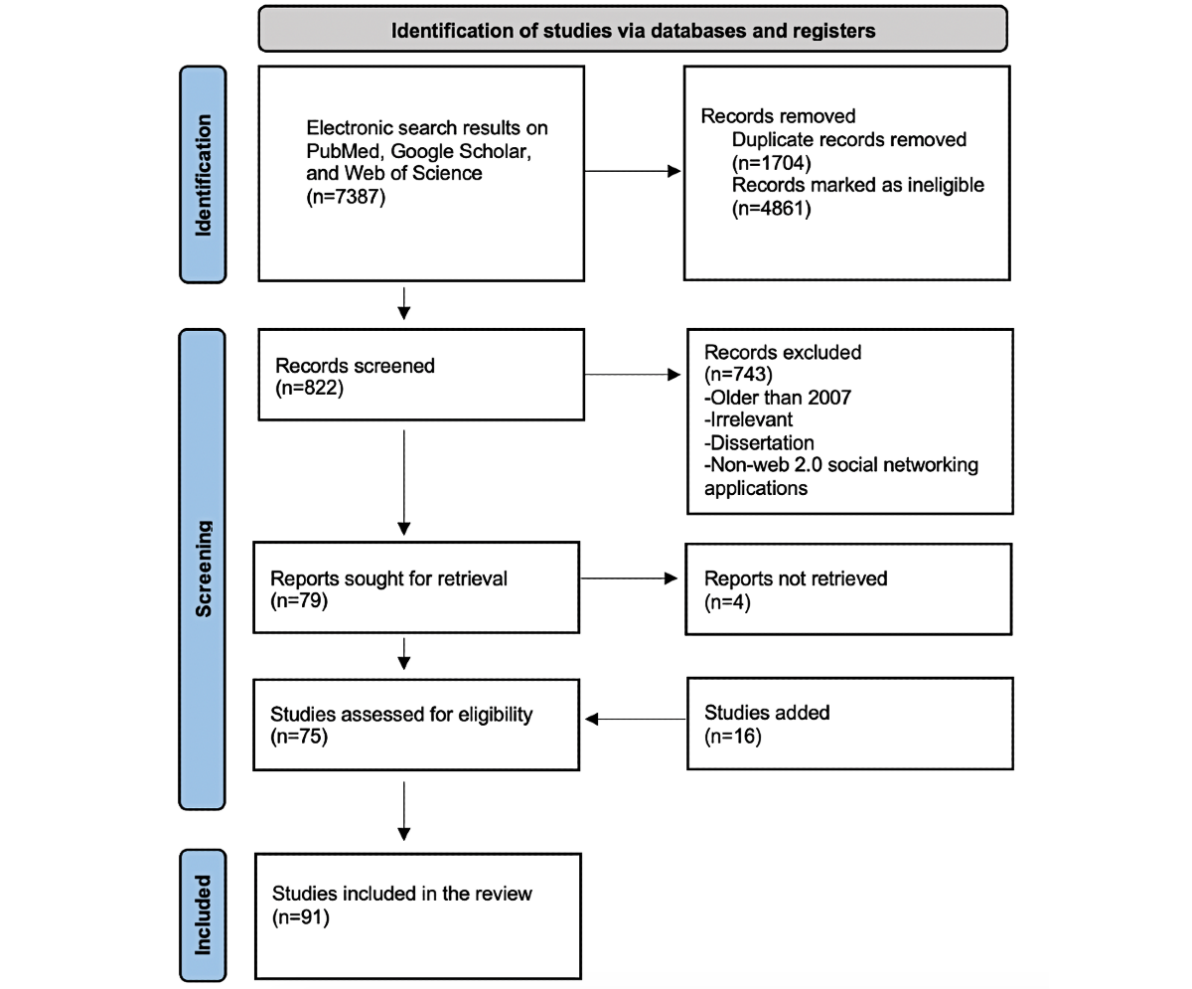
Flowchart of the literature search results.

A total of 7387 articles were retrieved from the search, and after removing the duplicate articles, 5683 (76.93%) articles remained. A total of 85.53% (4861/5683) of articles were marked as ineligible and were thus excluded. An additional 13.07% (743/5683) of articles were excluded after title and abstract screening based on the inclusion/exclusion criteria, and 0.07% (4/5683) were irretrievable. The full text of 1.31% (75/5683) of publications was screened and included. Owing to the daily emergence of relevant publications and reference screening, 16 more studies and 1 textbook chapter were added as the review proceeded by updating the search. A total of 91 articles and 1 textbook chapter were included in the analysis. Figure 1 shows a flow diagram explaining how the final inclusion was attained after the selection procedure.

### Characteristics of Included Studies

**Figure 2:**
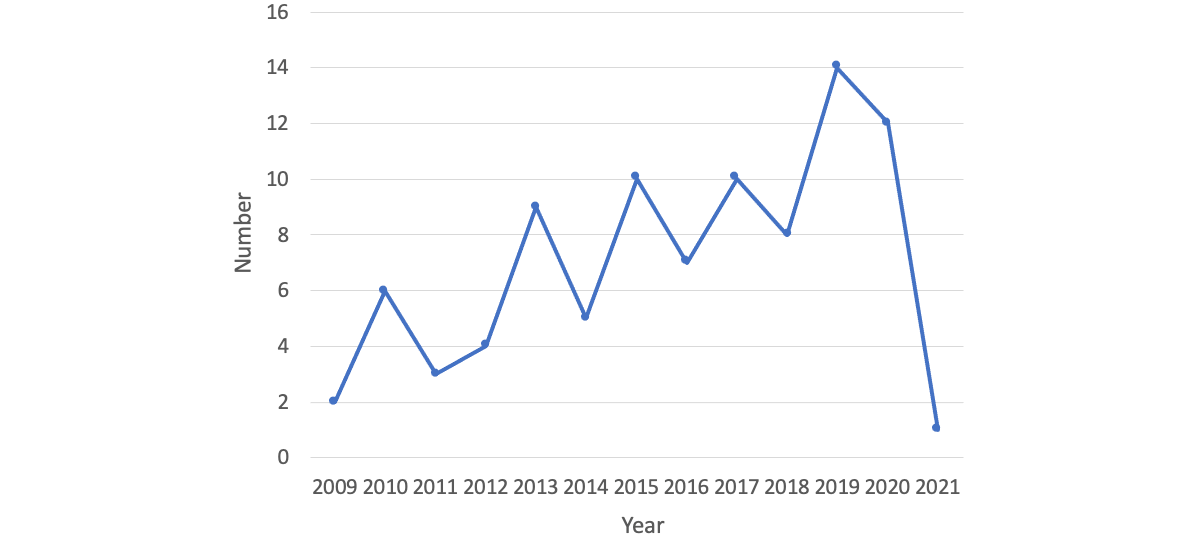
Number of included publications per year.

Figure 2 shows the number of included studies per publication year, with more than half of them published in the last 5 years. In terms of geographic location, the 91 publications were distributed as follows: 40 (43%) from the United States, 6 (6%) from Canada, 2 (2%) from Latin America, 10 (10%) from the United Kingdom, 13 (14%) from Europe, 8 (8%) from the Middle East, 1 (1%) from India, 7 (7%) from Asia, and 4 (4%) from Australia.

The included publications were complemented with web references and a textbook chapter. Original studies accounted for 42.8% (39/91) of the cited references. The remaining publications were meta-analyses, systematic reviews, narrative reviews, coping reviews, short communications, commentaries, viewpoint papers, and overviews. The social media platforms specifically investigated in some of the studies were Twitter or Weibo (n=1), WhatsApp or WeChat (n=10), Facebook (n=6), YouTube (n=2), Instagram (n=3), and blogs (n=1). Multimedia Appendix 1 [1-91] provides characteristics of the included 91 studies in chronological order.

### Qualitative Synthesis of the Results

All relevant information regarding the research question was extracted and summarized from the included studies. Information was then categorized into the emerging themes, as presented in the review: (1) social media use from the perspective of patients; (2) issues pertaining to the use of social media in health care; (3) ethical considerations; and (4) public health implications. The retrieved information was then qualitatively synthesized in the discussion for each category.

## Discussion

### Principal Findings

HCPs and patients typically represent the 2 ends of most health care relationships. HCP is a term used in this review to include physicians, dentists, nurses, medical or dental allied personnel, and health care organizations, whereas patients is a term used to include patients under the care of an HCP and the public. There is overlap in the ways HCPs and patients use social media. In the following section, only information unique to the perspective of patients, which has not been covered in Part I, is presented [11]. Collaterally during the search, studies that investigated ethical and legal considerations in the use of social media and others that discussed its shortcomings and barriers have emerged. These points have also been briefly discussed.

### Social Media Use From Patients’ Perspective

#### Overview

In this digital age, people are accustomed to using the internet for health communication. The new term *netizen* has been introduced and is informally used to describe a habitual user of the internet. It is indisputable that patients greatly incorporate social media in seeking health care and that the public is heavily reliant on it to obtain health care information. Perhaps no example supports this notation, as recently witnessed amid the COVID-19 pandemic. There is an abundance of information in the literature pertaining to this subject. In the following section, information has been presented in 5 categories.

#### Health Information

For a good proportion of the public, young people in particular, social networking sites are the first resource to find general and health-related information [1]. Many individuals with a medical concern are now seeking answers on the web and can virtually obtain them at anytime from anywhere [12]. Social media has radically transformed the way patients obtain information about procedures as well. In a 2009 study, 61% of American adults reported looking on the web for health information [13]. Another study in 2013 found that the first motive of patients for health-related use of social media is seeking information about health, a disease, or treatment of a disease; Twitter was the most commonly used platform for that information [14]. Moreover, 74.9% of web-based health-related information seekers searched for oral health–related information [15].

Health organizations, HCPs, and lay people make an exceedingly large amount of health-related information available on social media. However, the amount of information available may be overwhelming, and the sources may be unverified. The authenticity of the information posted should be questioned, and the recipients must be wary of the information they encounter because many posts do not undergo any quality regulation or verification, and the users are usually in control of the content they encounter [13].

Perhaps there has never been a time where social media was used to obtain health care information, as was the case during the COVID-19 pandemic. In a single day in March 2020, COVID-19–related terms were mentioned more than 20 million times on social media [92]. Almost every social media platform imaginable contributed to the dissemination of information pertaining to the pandemic. Health authorities have used their social media accounts to effectively share scientific information and combat what has been described as an infodemic [93]. Now that vaccines against SARS-CoV-2 are available, social media has been used again as a public podium for individuals to share their thoughts of and experiences with vaccination. Although social media has an unprecedented capacity to make evidence-based information accessible to the public and promote positive health behaviors, it has also been a major factor in propagating vaccination hesitancy, thus posing a threat to global public health [16,17].

In conclusion, HCPs will continue to be challenged by misinformation readily available to patients on social media. They must be determined to abide by evidence-based health care and ready when challenged by misinformed patients. HCPs also have a duty to make scientifically solid information more accessible to the public. At present, targeted health education interventions are strongly encouraged to foster public trust in vaccination and increase their uptake of the COVID-19 vaccine.

#### Telemedicine

Communication and monitoring in health care have been outsourced to social media in recent years. Appointments became web-based, health information became available on the internet, and examinations and laboratory results became available on the web-based portal of the facility [18]. Care has been delivered remotely through telemedicine apps, which are the best access to care for some populations, such as those in isolation or in rural areas [12]. Monitoring patients in their homes can improve health care services [19]. Good overall satisfaction has been reported with new telemedicine strategies that shift care to a more patient-centered one [9]. Not only is telemedicine efficient, but it is also time- and cost-saving.

In a 2016 study, telemedicine impression via WhatsApp and clinical assessments was consistent in 82% of the cases examined. Furthermore, telemedicine consultation reduced geographic barriers for initial clinical consultations, and most patients were encouraged to pursue a clinical examination [4]. For instance, Georgia Health Sciences University has enabled patients to access a web-based platform to reach their physicians to ask questions or request prescription refills [20]. There is evidence that telemonitoring of pregnancy is effective, especially for patients in rural areas who do not have to travel to a hospital [9]. In a 2018 study on telemedicine in China, a participant made a comment that suggested seeing a physician while staying at home if people could shop while staying at home [21].

To summarize, patients are encouraged to use telemedicine services that have become readily available and have remarkably improved since the COVID-19 pandemic. However, they must also remember that telemedicine is not the only means to receive health care, nor is it suitable for all cases. Patients have a right to traditional health care as needed and must comply with traditional appointments and hospital visits that are deemed necessary by the treating physician.

#### Finding an HCP

Social media has now become the new word of mouth. Web-based resources are being increasingly used and highly regarded to make health care decisions, including finding an HCP [6]. In fact, a considerable number of patients are currently searching for HCPs on social media. Some make educated decisions after comprehensive research on the academic qualifications and experience of the practitioner, whereas others follow their emotions after encountering an inviting post or an attractive image, with the latter comprising a huge pool of patients [6,22,23,94].

The content available on social media has an impact on prospective patients: 41% of social media users are influenced by the content they encounter on the web [95]. For example, a study showed that patients are keen to know qualifications of dentists before they visit the office and may use of LinkedIn for that purpose because many dentists showcase their expertise on that platform [24]. Furthermore, patients ranked academic qualifications as the most important content they sought on a Facebook page; some reported that they also sought positive reviews and awards in addition to the original content. In another study, patients reported that the most important factors in selecting a dentist on social media were the reviews and the qualifications of the dentist, with the least important factors being the awards obtained and the number of likes [22].

The attractiveness of a practitioner or provider on social media should not be underestimated. In fact, a study found that 57% of consumers thought that hospitals’ social media presence would strongly influence their hospital choice [25]. In another study, 53.4% strongly agreed about the necessity of having a social media presence for dental practices, and 55.1% thought that social media presence was effective in attracting new patients [22]. An interesting study on plastic surgery practices found that the average total number of followers per practice was significantly associated with the placement of the practice on the front page of Google, compared with the second page. Even after a multivariate adjustment of years of experience and education, use of social media remained an independent predictor of placement on the front page of a Google search [6]. A review by Nayak and Linkov [26] showed that patients used social media to find surgeons and that the social media presence of the surgeon can dramatically increase their image as an expert. On the other hand, it was found that unprofessional behavior of an HCP on social media can adversely affect the trust of patients [27,28].

Similar to most marketing strategies, there is no one-size-fits-all means to be successful as an HCP on social media. However, if HCPs recognize the importance of building a relationship with their audiences through social channels, their brands will become more credible and appealing to the target patients. On the other hand, patients must perform due diligence to profile HCP credentials and not rely solely on their perception of their presence on social media.

#### Peer Support and Sharing Experiences

Not only do HCPs find support and compassion on social media but also do patients. Individuals with chronic disease use social media to communicate with others and exchange experiences. This is especially helpful in rare medical conditions, in which case patients may be geographically distant. Even the family and friends of patients can receive emotional support or request guidance and advice from health care professionals on social media platforms.

Facebook groups for individuals with specific medical conditions are abundant and actively engage members in peer-to-peer support [29,30]. A number of social networking sites, such as *PatientsLikeMe*, provide patients with information and the opportunity to gain support from other people with the same medical condition [31]. Instagram accounts have also been created to provide information and peer-to-peer support for patients with health care needs, such as adolescents with type 1 diabetes [32]. Moreover, a study showed that a WhatsApp group for hypertensive patients with type 2 diabetes promoted the adherence of patients to treatment [33].

Health-promoting messages coming from social networks instead of experts were perceived as less disempowering and more effective [13,34]. YouTube has been used by patients with cancer to share personal stories [35]. Moreover, a recent study explored cancer survivorship on social media and found that the content shared by survivors displayed their physical, emotional, and psychological health [36]. Although Instagram was used mainly for sharing images posted by survivors themselves or others, Twitter was used primarily for sharing facts and fundraising. In the first week of the COVID-19 pandemic, Twitter users were found to use the tool to notify or warn their friends and followers about the outbreak; that is, Twitter was a platform for people to bond around the topic of COVID-19 [37].

Patient experience is receiving a substantial amount of attention lately, and social media provides patients with opportunities for their voices to be heard and their conversations to be amplified. They can share their experiences in discussion forums, via instant messaging, or post them on the web for the public to see [38]. As patient communities become more interconnected, patients can recommend or defame a practice and compare different experiences. Social media also allows patients to *like* posts, which may elicit notifications to others in their networks [39]. Word-of-mouth marketing between patients with similar conditions or circumstances is also easy with social media. Recommendations or opinions of users have been perceived to be more credible than other advertisement methods, mainly because of the personal nature of the communication that takes place between users on social media [13].

In conclusion, patients find support from peers on social media and express their feelings about their well-being and the health care they receive. It seems that a snowball effect occurs in patient communities on social media, where the more patient-generated content is being shared, the more the public is attracted, the more interaction takes place, and the more content is generated in return.

#### Influencing Positive Health Behavior

Supplemental electronic communication with patients has been found to emphasize health care guidelines and improve treatment adherence in patients with chronic diseases [40]. In 1 study, 60% of physicians reported favoring interacting with patients on social media to encourage behavioral changes and drug adherence in the hope that these efforts would lead to better health outcomes [41]. Through social media platforms, HCPs can disseminate positive messages to a wide population of users swiftly and influence healthier behaviors through social reinforcement [42]. For example, a study used several social media platforms to encourage blood donation, indicating that social media helped to improve blood donation practices in Saudi Arabia, where there is a shortage of blood donors [2]. Furthermore, a 23-fold increase in donor pledge in web-based state organ-donor registries was observed just a week after Facebook allowed its users to state their organ-donor status in their profile [42]. A review by van den Heuvel et al [9] found that exercise apps possibly led to less gestational weight gain and an increase in smoking abstinence in pregnant women.

Social media can also increase the public’s awareness and compassion toward individuals with special health care needs. Social media platforms are increasingly being used for antistigma campaigns to influence public attitudes. Having their unheard voices made public without barriers can be of tremendous relief to individuals with special health care needs. An example is the role of social media in destigmatizing epilepsy [43]. Moreover, Twitter has been successfully used to combat mental illness stereotypes. The platform has facilitated education and contact between individuals with mental illness and has also highlighted injustice [44]. Facebook also enables users to discuss mental illness without the burden of social discomfort [44]. In China, where sharing the intention to attempt suicide on social media is considered a public health concern, social media can be successfully used to enhance suicide literacy and thus be effective for reducing the stigma attached to suicidal ideation and increasing help-seeking behaviors [45]. In Australia, social media is considered an effective means of delivering suicide prevention activities to a large number of young adults [46]. A project called #chatsafe was developed to assist young people in communicating about suicide via social media to feel better and deglorify suicide; the project was recently globalized [47,48].

Just as social media has the potential to promote healthy behaviors, it can also reduce risky behaviors. It can expand the reach of public health efforts and deliver intervention content in an interactive format. An example is smoking cessation campaigns [49]. Reminders and discussions on Facebook and WhatsApp were found to be effective in preventing smoking relapse in individuals who had stopped smoking [50]. In a 2017 systematic review, Facebook and Twitter were found to be feasible and preliminarily effective for smoking cessation, with studies reporting greater abstinence, reduction in relapse, and an increase in quitting attempts among users [10]. These findings are in agreement with the results of a more recent review, in which the use of Facebook, Twitter, and WhatsApp by an online smoking cessation community showed promising results in helping smokers quit [51]. An initiative on Facebook targeted young adults as an intervention for smoking and heavy drinking [52]. Although the interest in changing smoking habits was bigger than that for drinking behavior, and the participants favored changing 1 habit at a time, they accepted and received the post messages well. In a review by Kazemi et al [53], social media was found to help provide HCPs with a platform for combating illicit drug use. It was also found that social media can identify patterns of emerging drug use and that data mining tools can complement the current surveillance methods for tracking drug abuse. In a 2019 cross-sectional study, Generation Z and millennials, a population with high rates of substance use disorder, thought that social media platforms could be helpful in preventing recurrent drug use; however, fewer than half of the participants expressed a willingness to be monitored via social media to support their recovery [54]. Participants from both cohorts had seen more drug cues on social media than they saw recovery information, which highlights the need for digital interventions to improve drug use treatment and recovery outcomes.

The impact of social media on sexual behavior has also been investigated. One study created an intervention page on Facebook to promote sexual health and serve as a safe space for youth to share ideas and experiences with peers and professionals [55]. It was reported that for a short term (baseline to 2 months), condom use among high-risk youth in the intervention group was stable, whereas it decreased in the control group. Furthermore, the Facebook initiative was able to reach minority communities in which sexually transmitted infections and HIV infections were prevalent. In a 2016 review, 51 studies that investigated social media for sexual health promotion with social media as the sole intervention or in combination with other interventions were reviewed [56]. A total of 8 publications reported increased condom use, use of health services, and HIV self-testing. Two publications reported a reduction in gonorrhea cases and an increase in syphilis testing. Most publications targeted the youth. Facebook is the most commonly used social media platform, either exclusively or in conjunction with other platforms.

There is evidence that social media promotes physical activity and weight loss. In China, a study compared weight loss among participants in a control group (receiving routine publicity on weight loss) and those in a WeChat group with 6 months of weight loss intervention [3]. Male participants in the WeChat group lost significantly more weight than their control peers, although the former were significantly younger. It was found that the more actively participants were using WeChat, the more weight they lost. Another study among medical students found that those who were part of a motivational Facebook group increased their physical activity after 1 month. The likelihood (odds ratio) of becoming sufficiently active by joining the Facebook page was 3.51 [57]. A study on 341 college students with obesity found that the social media approach facilitated short-term weight loss, with the participants losing considerable weight at 6 and 18 months [58]. An initiative on Instagram was found to be attractive and effective in reinforcing the maintenance of an appropriate level of physical activity [59]. In another study, a health app was developed and found to be successful in motivating users to be physically and socially active in real life [60]. During the COVID-19 pandemic, videos of trainers motivating people to work out in their homes during the lockdown went viral. Similar initiatives were seen taking place on every continent, and what could have been a depressive sedentary lockdown to many became a more bearable time.

Cancer prevention efforts have traditionally focused on adults. As health behaviors can aid in cancer prevention, and many behaviors are established in young adulthood, it is logical to target preventive programs in the younger population. In addition, because most of today’s youth are digital natives, using social media for promoting cancer-preventing behaviors seems to be a promising strategy. A comprehensive study discussed the potential of social media in cancer prevention and laid the foundation for future research [61].

A comprehensive 2019 systematic review found variation in the strength of evidence regarding the impact of social media on behavior change [96]. However, social media campaigns have generally aided in the reduction of sedentary behavior, contribution to smoking cessation, and improved sexual health, in addition to being cost-effective. It was also found that social media better prompted users to access support services, especially smoking quit phone services. Illicit drug and smoking campaigns appeared to be more effective for the younger generation. Furthermore, expanding the duration or intensifying campaigns was found to be effective. Evidence suggests that targeting messages at a specific target audience increases their impact.

In conclusion, social media has helped patients adhere to treatment, access health care guidelines, and adopt positive health habits to varying degrees. There is no single platform for obtaining these positive outcomes. Stakeholders, researchers, and HCPs must use the platform they consider more effective for and accessible by their target population and customize their content in terms of simplicity, frequency, method, and duration. Researchers should aim to conduct studies that can be effectively adapted to more than one platform or setting and reach a larger population. Future studies should include greater racial diversity among the participants.

### Issues Pertaining to Social Media Use in Health Care

There will always be a positive and a negative side of using social media in health care [62]. Although social media has been heavily used by health organizations, medical personnel, patients, and the public, in general, its use is associated with barriers, limitations, and shortcomings. First, internet connectivity is required to access social media. Despite the widespread use of the internet worldwide, 41% of the global population still has no access to the internet [97]. Unfortunately, low-income families and individuals with disabilities are less likely to use the internet, resulting in further exclusion of individuals who are already marginalized [63] Second, some degree of technology skills is essential to enter the digital world. Although basic skills are not very difficult to acquire, digital literacy can be challenging for some populations, such as older adults and individuals with intellectual impairment [64].

Some studies have investigated the shortcomings of technology-mediated remote health care. Inefficiency of web-based medical visits compared with face-to-face engagements has been perceived [65]. A dermatology study found that the quality of the images obtained in group discussions was inconsistent [66]. There is also a fear that patients enjoying the convenience of telemedicine are deterred from visits to the hospital when necessary [14,67]. Moreover, financial limitations should be considered since e-consultations and web-based visits may not be covered by insurance companies [14].

Connections established through social media may dissolve the boundaries between professional and personal lives [68]. A recent study found that patients often extend internet *friend* requests to their physicians on Facebook; however, recommendations often discourage personal web-based communication between practitioners and patients [40]. Personal boundaries may be violated by inappropriate curiosity, as social media can provide a wealth of information about its users [25,69]. Patients may have unrestricted access to the personal information of HCPs available on the internet, and HCPs also have access to patient information that may not be available in the health care setting. Nevertheless, patient information received from web-based sources may be helpful in certain health care settings; for example, HCPs may observe a lack of adherence to medical recommendations and may alter management accordingly [18].

In social media communication between patients and HCPs, there may be frequent interruptions; the false sense of *having to* be available 24/7; disparity on urgency; compromised verbal communication and body language, especially in texting services; noncompliance with specific terms of a social media platform; lack of proper guidelines for group moderators to manage discussions and controlling content; difficulty in obtaining printed records of communication; and no accurate records of all web-based encounters in the medical records of the patients [27,70,71]. There is also the possibility of identity theft, since any user can create an account, use any name and profile picture, and claim to be someone else. For instance, the logo of the American Society of Colon and Rectal Surgeons was used by a hospital in a different country to request an endorsement [8].

Social media is a double-edged sword for HCPs. As fast as a positive review travels, so does a negative one. Patients unhappy with a service, payment, treatment outcome, or legal actions may start a war against the practitioner or practice. Teaming up with more keyboard warriors or internet trolls can have a disastrous emotional and professional impact on HCPs. In 2016, a well-respected orthopedic surgeon was awarded US $480,000 in damages for defamation after continual vilification by a patient and her kin through a website and social media. The defamatory material included a fake shaming website that greatly resembled the legitimate business website of the surgeon, on which they referred to him as *the butcher*. Similar materials were posted on a couple of social media platforms such as Facebook, YouTube, and Pinterest [98].

HCPs usually support and defend one another. However, some may find social media a good medium to begin a battle against a competing HCP, justifiable or not. Negative professional criticism, displayed publicly on social media, is a violation of the medical codes of ethics; it expresses ill will and aims to tarnish the image of one’s professional colleagues. Destructive negative criticism of colleagues on social media damages the medical profession and its reputation. On a positive note, digital shaming is unlawful in many countries and may lead to legal consequences [99].

Although it comes at a relatively low cost, the volume of information on social media may be overwhelming. In addition, the information can be unreliable, difficult to prove as valid, vary in quality and consistency, outdated, not subjected to peer review, invalid, incorrect, not applicable to all situations, not generalizable, opinions and preferences presented as facts, or entirely false [14,38,72]. This is a public health threat, the effect of which is difficult to quantify. It can be difficult for inexperienced HCPs and the public to discern reliable information; thus, there is a risk of absorbing both valid and less credible information. With digital media, social media in particular, misinformation can be easily amplified within echo chambers, which consist of individuals with similar mindsets and beliefs [73]. With artificial intelligence incorporated into technology, algorithm-driven filters selectively display content based on user preferences [73]. For example, a mother who is uncertain about vaccinating her child may join a group of antivaccine mothers to learn more about their concerns. Not only would she be bombarded with antivaccination information, from that point on, antivaccination related information will be targeting her on several social media platforms, fostering antivaccination which may not be at her nor her child’s best interest.

It is a fact that public voices disseminating inaccurate health information are usually far better heard and related to than evidence-based knowledge from experts and official health organizations [74]. It was noted that disinformation travels at the same speed that information does, which is why some organizations and authorities have dedicated time and effort to fight myths and disinformation in social media platforms, as seen in the exclusive website section of the World Health Organization dedicated to myth-busting COVID-19 disinformation [72,75]. Another negative consequence of social media is the poorly defined audience; information shared by HCPs may entirely miss the target population. Moreover, with social media, there is a risk of early adoption of unvalidated research and preliminary findings that carry a risk of future medical reversal, which would create more hesitancy in the public and HCPs alike [73]. Another major problem in publishing scientific information on the web is that the user may have hidden conflicts of interest that are not disclosed. It is crucial that every effort be made to critically appraise the information available on social media.

The rapid speed at which information travels may have a very negative impact on the general well-being of the public. For example, disseminating alarming and exaggerated information, misinformation, and manipulated information about COVID-19 may cause fear, anxiety, undue stress, and depression at a societal level, even in individuals without underlying psychiatric illnesses [72]. People may also publicly share their negative feelings, such as anxiety, worry, and conspiracism on social media. Such posts may have a contagious effect. At the beginning of the COVID-19 pandemic in the first few weeks of 2020, a study in China surveyed over 4000 participants. Frequent exposure to social media was associated with high odds of anxiety and depression in the general population as well as among health care workers [5]. Another study found that 53.8% of respondents expressed encountering a moderate or severe psychological impact from the COVID-19 pandemic [76]. Furthermore, a UK study found a positive relationship between the use of social media as a source of information on COVID-19 and conspiracy theory beliefs, especially among younger participants [77].

Being highly influential and used by a large young population, social media may also promote unhealthy habits such as tobacco and alcohol use, violence, unhealthy dietary choices, and high-risk sex, especially if they are promoted by digital community leaders (ie, influencers) [70,78,79]. Furthermore, enforced advertisement on social media and the subconscious messages of what *looks* good through seductive photographs may have negative unintended consequences for body image and self-esteem in some users and could provide patients with unrealistic expectations for treatment [80]. The public is usually unaware that practitioners showcase successful outcomes selectively and that the pictures may not reflect the true skills and proficiency of a practitioner [71]. This may discourage students and recent graduates who may have not yet obtained the skills of experienced HCPs. Some social media groups are based on misconceptions and can be misleading to the public, such as groups that promote freedom to take off the masking during the COVID-19 pandemic. However, social media platforms have begun taking action to limit discussions of that sort [74].

Posting photographs of procedures and *before-and-after* photographs in a reasonable amount may be beneficial and educational; however, some practitioners make it a goal in itself. If overdone, these posts lose their educational value and become unprofessional advertising and marketing tools [80]. In addition, the pressure to be socially accepted and celebrated, especially through social media, may be difficult to handle. Some individuals, including HCPs, measure their self-worth and seek validation from feedback on social media (eg, number of followers, retweets, and likes). Social media users whose self-confidence is lacking can become more anxious or depressed, which will lead to less self-confidence and erosion of self-worth. It is advisable that HCPs re-evaluate the value of social media if it starts to affect them negatively. It might be advisable to cut back or opt-out all together. Just as it applies to the public, if HCPs are psychologically impacted and struggling, it is better to seek professional help early on.

Although the use of social media among adolescent patients has been shown to be effective in promoting positive health behaviors such as increased physical activity and smoking cessation, the negative impact of social media on the mental health of young people cannot be neglected [50,60]. There is evidence to support less use of social media as a protective factor for mental health in young people [81,82]. In recent years, cyberbullying has emerged as a threat to the mental well-being of young people. A 2015 review found a consistent relationship between cyberbullying and depression among adolescents [83]. In another review, victims of cyberbullying were found to be affected by worry, fear, depression, and loneliness [7]. It was also found that being a cyberbullying victim was associated with more self-injurious behaviors and suicidal thoughts. In the 2019 study by Viner et al [84], the authors analyzed data from the Longitudinal Study of Young People in England and found that the frequent use of social media by young girls was associated with decreased well-being and increased psychological distress. However, they also found that the negative impact of using social media appears to stem from the harmful content users are exposed to and the displacement of healthy lifestyles rather than social media use per se. A review in 2017 found that social media use substituted social interactions, leading to depression and anxiety [7].

A major problem with social media use is that the content posted is prone to be judged and evaluated by whoever sees it. The judgment can be very subjective based on the rater and may reflect unfavorably on HCPs. The trust of patients may be shaken over one *bad* or *inappropriate* post. There are no clear guidelines about e-professionalism and what is considered appropriate; it is inherently subjective [85,86]. A review by Neville and Waylen [27] displays practical examples of e-professionalism that help simplify the concept. The digital footprint has an impact not only on the reputation of the user but also on the profession. Postings on social media can be a permanent record, even after the content is deleted.

Social media posts can be viewed by a large audience base beyond the intention or imagination of users [38]. Employers, program directors, and health officials have the authority to discipline HCPs for unprofessional behavior or breaches of patient privacy, which may ultimately affect the credentials and licensure of the practitioners [20,40,87]. Even appropriate posts may be unfairly scrutinized and negatively judged when viewed out of context. There is also the problem of conflicting timestamps, such as a tweet or a post shared at a time when the HCP was in the middle of a procedure or should have given greater attention to a clinical situation, which could be very damaging to a jury of peers and the public’s opinion [8].

In the United Kingdom, 45% of pharmacy students stated that they have posted content on the web about which they are not comfortable with future employers seeing [88]. In addition, about 60% of medical schools reported incidents in which students posted inappropriate content on the web [20]. Furthermore, over half of the medical students surveyed in one study admitted to having embarrassing Facebook photographs of themselves [89]. In a study by Langenfeld et al [86], 12.2% of residents had had clearly unprofessional behavior on Facebook, such as Health Insurance Portability and Accountability Act violations and binge drinking; an additional 14.1% demonstrated potentially unprofessional behavior, including political statements and the use of alcohol and tobacco [86].

### Ethical Considerations

Social media communications with or about patients can lead to a breach of privacy and anonymity of patients, which may result in legal actions against HCPs and their institutions. To avoid legal consequences, any post about patients, whether in text, video, or image, should be deidentified, in accordance with Health Insurance Portability and Accountability Act regulations [25]. It is advisable to always obtain consent before sharing any patient information, even if the content is anonymized [71,90]. In 2011, an emergency physician discussed patient care on Facebook. Although she did not identify the patient, she shared enough information to make identification easy to others in her community. As a result, she was fired [100]. In 2016, a pediatric anesthesiologist made inappropriate political comments on Facebook and was ultimately fired from the University of Colorado [101].

It is paramount that HCPs realize that professional demeanor is expected on the internet as in real life. Although no formal contract is established between HCPs and patients in the web-based world, the same rights and responsibilities traditionally applied should be considered on the internet. In 2013, an obstetrician made unsympathetic comments about an always late patient. She accidently made them public. The post and subsequent comments became viral and was featured on the news. Thousands of people petitioned, and the physician endured several professional and personal consequences, but she was not fired from her practice [102]. In another instance, a patient complained to the media about a hospital in California; in retaliation, the hospital disclosed information about the patient to the media without permission and was ultimately fined US $275,000 [103]. In April 2020, an emergency physician in Washington was fired after criticizing his hospital for its COVID-19 response on social media [104].

There are several other issues pertaining to ethical considerations when using social media in health care. One example is the recruitment of minors on social media for research purposes. It is not difficult to locate and recruit research participants below the age of 18 years on social media. However, individuals below that age have not reached cognitive maturity to make thought-through decisions regarding participation in research. Obtaining parental consent or targeting parents may be a more ethical alternative [18]. Another example is falsifying images posted on social media. Photographic technique artifices, such as modifying angles or digitally altering photographs to exaggerate treatment outcomes, is deceiving to patients and is considered unethical abuse [80].

As the use of social media by HCPs has increased, health authorities have published guidelines and recommendations for the use of social media. For example, in 2011, the American Medical Association published its policy on professionalism on social media [91]. Later in 2013, the General Dental Council in the United Kingdom published a document titled, *Guidance on using social media* [105]. It is imperative that medical curricula tackle e-professionalism, professional internet etiquette, and digital ethics, as the use of social media in health care is the new norm among the millennial generation of HCPs. For more information, it is recommended to read the review by Langenfeld and Batra [8], in which recommendations for e-professionalism have been proposed. In addition, refer to the guidelines on the use of social media that have been summarized by Dhar [71].

### Public Health Implications

Social media has the potential to transmit health-related information and promote health to the public. Striking the right balance between digital and traditional health care is imperative. Social media is omnipresent in our lives today, and the best guard we have is to be acquainted with it and practice due diligence in using it to our favor for the promotion of health care. Nevertheless, HCPs, patients, and the public in general need not forget the risks to which they may be exposing themselves. As medical professionals, HCPs are bound to ethical principles toward their colleagues, patients, and the public in the digital as much as in the real world. Whether e-professionalism is formally taught, ethics is a matter of choice.

### Limitations

Despite its comprehensiveness, because of this review being a narrative review, it is descriptive in nature and did not include a formal appraisal of the included studies. Data from the included studies were summarized and reorganized but not analyzed. Although our search was comprehensive, some relevant studies may have been unidentified. Bias may have occurred in selecting and assessing the literature, as it was not done in a systematic manner, giving the type of review.

### Conclusions

This narrative review aimed to discuss how patients have been using social media in the context of health care and describe the main issues pertaining to its use in health care. As can be seen, multidimensional health care, such as when pairing health care with social media and other forms of communication, has been shown to be very successful. The outcome is maximized when the audience is reached numerous times, in multiple settings, and from various sources. The number of digital natives is increasing and will continue to grow in health care settings. Thus, it is advisable to acknowledge that social media will remain an essential part of health care for many years.

Despite emerging evidence that the use of social media has facilitated health care, it has not and will probably not entirely replace traditional health care. The use of social media is associated with barriers, limitations, and shortcomings that continue to emerge in the literature. To maximize the benefits while minimizing compromise to the care provided and avoiding liability, HCPs and patients must perform due diligence before considering social media in health care and should make educated judgments on a case-by-case basis.

As social media is a relatively recent occurrence, more research is needed to determine its long-term effectiveness and to find the best strategies that would maximize its advantages while limiting its risks. e-Professionalism and the ethical considerations in using social media in health care can be further explored.

## Appendix

Multimedia Appendix 1 Characteristics of the included 91 studies in a chronological order.

## Abbreviations

HCP: health care provider
MeSH: Medical Subject Headings
