# Supplementary material for: Social Media and Health Care (Part II): Narrative Review of Social Media Use by Patients
Source: J Med Internet Res. 2022 Jan 7;24(1):e30379. doi: 10.2196/30379 (PMC8783277; doi:10.2196/30379)
Supplement: Multimedia Appendix 1 [file jmir_v24i1e30379_app1.docx]

**Multimedia Appendix 1. Characteristics of the included 91 studies in a chronological order.**

| **Author** | **Year** | **Title** | **Country** | **Type of Evidence ^a^** | **Main Objective/s** |
| --- | --- | --- | --- | --- | --- |
| Cain and Romanelli | 2009 | E-professionalism: a new paradigm for a digital age | US | Secondary | To define and elaborate on a new construct of e-professionalism. |
| Kim | 2009 | Content analysis of cancer blog posts | US | Primary | To explore the efficacy of user-defined subject tagging and software-generated subject tagging for describing and organizing cancer blog contents. |
| Chretien et al | 2010 | It's your own risk: medical students' perspectives on online professionalism | US | Primary | To explore medical student perspectives on online posting. |
| Clauson et al | 2010 | Use of blogs by pharmacists | US | Primary | To examine the characteristics of pharmacist blogs. |
| Garner and O'Sullivan | 2010 | Facebook and the professional behaviours of undergraduate medical students | US | Primary | To investigate how undergraduate medical students use the social network website Facebook, and to identify any unprofessional behaviour displayed online. |
| Kreps and Neuhauser | 2010 | New directions in eHealth communication: opportunities and challenges | US | Secondary | To review key communication issues involved in the design of effective and humane eHealth applications. |
| Levac and  O’Sullivan | 2010 | Social Media and its Use in Health Promotion | Canada | Secondary | To provide information about the possibilities of using social media to improve access to health information and health care providers. |
| Lo et al | 2010 | YouTube: a gauge of public perception and awareness surrounding epilepsy | Canada | Primary | To determine how epilepsy is being perceived on a video-sharing website. |
| Bosslet et al | 2011 | The patient-doctor relationship and online social networks: results of a national survey | US | Primary | To quantify the use of online social networks, patient interactions within these networks, and attitudes toward them among medical students, resident physicians, and practicing physicians. |
| Chou et al | 2011 | Cancer survivorship in the age of YouTube and social media: a narrative analysis | US | Secondary | To provide an in-depth description of authentic personal cancer stories. |
| Shore et al | 2011 | Report of the AMA Council on Ethical and Judicial Affairs: professionalism in the use of social media | US | Secondary | To discuss the ethical implications of physicians' nonclinical use of the internet, including the use of social networking sites, blogs, and other means to post content online. |
| Bull et al | 2012 | Social media-delivered sexual health intervention: a cluster randomized controlled trial | US | Primary | To determine whether sexually transmitted infections prevention messages delivered via Facebook are efficacious in preventing increases in sexual risk behavior. |
| Chauhan et al | 2012 | Social media and you: what every physician needs to know | US | Secondary | To discuss the risks of using social media by physicians.^b^ |
| Childs and Martin | 2012 | Social media profiles: striking the right balance | US | Secondary | To describe the advantages, disadvantage, the risks and benefits in social media use in healthcare. ^b^ |
| Lambert et al | 2012 | Risk management and legal issues with the use of social media in the healthcare setting | US | Secondary | To discuss risk management and legal issues with the use of social media in healthcare. ^b^ |
| Antheunis et al | 2013 | Patients' and health professionals' use of social media in health care: motives, barriers and expectations | Europe | Primary | To investigate patients' and health professionals' motives and use of social media for health-related reasons, and barriers and expectations for health-related social media use. |
| Farnan et al | 2013 | Online medical professionalism: patient and public relationships: policy statement from the American College of Physicians and the Federation of State Medical Boards | US | Secondary | To provide a policy for the best practices to inform standards for the professional conduct of physicians in the digital environment. |
| Fogelson | 2013 | Beyond likes and tweets: an in-depth look at the physician social media landscape | US | Primary | To provide background on social media communication, as well as specific advice for online physician communication and a discussion of confidentiality. ^b^ |
| George et al | 2013 | Dangers and opportunities for social media in medicine | US | Secondary | To summarize the hazards of social media in medicine and describe the most promising avenues through which professionals can use social media in medicine. |
| Hall et al | 2013 | Use and views on social networking sites of pharmacy students in the United kingdom | UK | Primary | To investigate students' use and views on social networking sites and assess differences in attitudes between genders and years in the program. |
| Househ | 2013 | The use of social media in healthcare: organizational, clinical, and patient perspectives | Middle East | Secondary | to explore the impacts of social media on healthcare organizations, clinicians, and patients. |
| Laz and Berenson | 2013 | Racial and ethnic disparities in internet use for seeking health information among young women | US | Primary | To examine the influence of race/ethnicity on seeking health information from the internet among women. |
| MacMillan | 2013 | Social media revolution and blurring of professional boundaries. | Canada | Secondary | To describe concerns in terms of human resource management, litigation and / or fines from various regulatory agencies for the provider and their employer. ^b^ |
| Moorhead et al | 2013 | A new dimension of health care: systematic review of the uses, benefits, and limitations of social media for health communication | UK | Secondary | To identify the uses, benefits, and limitations of social media for health communication among the general public, patients, and health professionals, and identify current gaps in the literature to provide recommendations for future health communication research. |
| Cairns et al | 2014 | Risk and protective factors for depression that adolescents can modify: a systematic review and meta-analysis of longitudinal studies | Australia | Secondary | To identify risk and protective factors for depression during adolescence that are modifiable by the young person. |
| Grajales et al | 2014 | Social media: a review and tutorial of applications in medicine and health care | Canada | Secondary | To present case studies that illustrate how, where, and why social media are being used in the medical and health care sectors. |
| Jain and Bickham | 2014 | Adolescent health literacy and the Internet: challenges and opportunities | US | Secondary | To explore challenges that adolescents face when using the Internet to access health information and opportunities for intervention. |
| Langenfeld | 2014 | An assessment of unprofessional behavior among surgical residents on Facebook: a warning of the dangers of social media | US | Primary | To evaluate the publically available Facebook profiles of surgical residents to determine the incidence and degree of unprofessional conduct. |
| Ventola | 2014 | Social media and health care professionals: benefits, risks, and best practices | US | Secondary | To describe benefits and risks of social media use in healthcare and suggest best practices. ^b^ |
| Betton et al | 2015 | The role of social media in reducing stigma and discrimination | UK | Secondary | To explore the implications of social media practices whereby people with mental health problems share their experiences in online public spaces. |
| Cheung et al | 2015 | Using WhatsApp and Facebook Online Social Groups for Smoking Relapse Prevention for Recent Quitters: A Pilot Pragmatic Cluster Randomized Controlled Trial | Asia | Primary | To determine if the group discussion and reminders via the WhatsApp or Facebook social group were effective to prevent smoking relapse in quitters who had stopped smoking recently. |
| Denecke et al | 2015 | Ethical Issues of Social Media Usage in Healthcare | Europe | Secondary | To summarize the ethical issues to be considered when social media is exploited in healthcare contexts. |
| Eisingerich et al | 2015 | Why recommend a brand face-to-face but not on Facebook? How word-of-mouth on online social sites differs from traditional word-of-mouth | UK | Secondary | To examine the conceptual difference between consumer electronic word-of-mouth on online social sites. |
| Hamm et al | 2015 | Prevalence and Effect of Cyberbullying on Children and Young People: A Scoping Review of Social Media Studies | Canada | Secondary | To review existing publications that examine the health-related effects of cyberbullying via social media among children and adolescents. |
| Neville and Waylen | 2015 | Social media and dentistry: some reflections on e-professionalism | UK | Secondary | To explore the various ways social media can help and hinder the practice of dental professionalism. |
| Robinson et al | 2015 | Social media and suicide prevention: findings from a stakeholder survey | Australia | Primary | To explore the ways in which stakeholders use social media for suicide prevention and assess their views about the potential utility of social media as a suicide prevention tool. |
| Saavedra Ramirez | 2015 | Social networks as a means of monitoring patients with hypertension and diabetes success story | Latin America | Primary | To identify the relationship between the use of WhastApp and adherence to self-care practices to patients in a specific hospital. |
| Thota and Divatia | 2015 | WhatsApp: What an App! | India | Secondary | To discuss a case in which the use of WhatsApp provided a vital role in transmitting medical information and images in a resource-limited situation. ^b^ |
| Yi-Frazier et al | 2015 | Using Instagram as a Modified Application of Photovoice for Storytelling and Sharing in Adolescents With Type 1 Diabetes | US | Primary | To assess feasibility markers for using Instagram for storytelling and sharing in adolescents with type 1 diabetes and examine and categorize the type of diabetes-related photos that would be shared |
| Al-Eisa et al | 2016 | Effect of Motivation by "Instagram" on Adherence to Physical Activity among Female College Students | Middle East | Primary | To investigate the efficacy of using Instagram with a home-exercise program as a motivational stimulus in improving physical activity adherence levels among female college students. |
| Almaiman et al | 2016 | Promoting Oral Health Using Social Media Platforms: Seeking Arabic Online Oral Health Related Information | Middle East | Primary | to estimate the prevalence of social media users among the Saudi population and identify the preferred social media platform for seeking Arabic oral health related information and its impact on seekers' knowledge, attitude, and behavior |
| Boulos et al | 2016 | Instagram and WhatsApp in Health and Healthcare: An Overview | UK | Secondary | To present an overview of the various applications of Instagram and WhatsApp in healthcare and to briefly describe the main issues surrounding the uses of these two apps in health and medicine. |
| Godino et al | 2016 | Using social and mobile tools for weight loss in overweight and obese young adults (Project SMART): a 2 year, parallel-group, randomised, controlled trial | US | Primary | To assess the efficacy of a two-year, theory-based weight-loss intervention that was remotely and adaptively delivered via integrated user-experiences with 6 different interventions. |
| Kaliyadan et al | 2016 | What's up dermatology? A pilot survey of the use of WhatsApp in dermatology practice and case discussion among members of WhatsApp dermatology groups | Middle East | Primary | To evaluate the frequency and types of uses, advantages and disadvantages of WhatsApp based clinical discussion groups as perceived by members of a few such groups. |
| Klaassen et al | 2016 | Usability in telemedicine systems-A literature survey | Europe | Secondary | To investigate how usability methods are applied in developing telemedicine systems. |
| Petruzzi and De Benedittis | 2016 | WhatsApp: a telemedicine platform for facilitating remote oral medicine consultation and improving clinical examinations | Europe | Primary | To describe use of the smartphone-based application WhatsApp to share clinical oral medicine information. |
| Barreto and Whitehair | 2017 | Social Media and Web Presence for Patients and Professionals: Evolving Trends and Implications for Practice | US | Secondary | To present the importance of social media  to health care providers and gives guidance on how to use it properly to maximize the positive effects and how to avoid common mistakes and optimize participation in social media. |
| Chaet et al | 2017 | Ethical practice in Telehealth and Telemedicine | US | Secondary | To provide guidance for ethical conduct relating to key issues in telehealth/telemedicine. |
| Golder et al | 2017 | Attitudes Toward the Ethics of Research Using Social Media: A Systematic Review | UK | Secondary | To ascertain attitudes on the ethical considerations of using social media as a data source for research as expressed by social media users and researchers. |
| He et al | 2017 | Social Media-Promoted Weight Loss Among an Occupational Population: Cohort Study Using a WeChat Mobile Phone App-Based Campaign | Asia | Primary | To evaluate the effectiveness of a mobile app as an intervention on weight loss behavior. |
| Hoge et al | 2017 | Digital Media, Anxiety, and Depression in Children | US | Secondary | To summarize emerging areas in the field concerning the relationship between digital media use and symptoms of anxiety and depression in children and adolescents. |
| Kazemi et al | 2017 | Systematic review of surveillance by social media platforms for illicit drug use | US | Secondary | To describe the ability of social media in recognizing illicit drug use trends. |
| Langenfeld and Batra | 2017 | How Can Social Media Get Us in Trouble? | US | Secondary | To review the dangers of social media and illustrate this with examples of unprofessional behavior and the associated consequences and to provide recommendations for maintaining a professional and productive online persona. |
| Muntaner-Mas et al | 2017 | Effects of a Whatsapp-delivered physical activity intervention to enhance health-related physical fitness components and cardiovascular disease risk factors in older adults | Europe | Primary | To evaluate the feasibility and preliminary effectiveness of a 10-week WhatsApp-based intervention aimed at enhancing health-related physical fitness components and cardiovascular disease risk factors compared with a face-to-face condition. |
| Naslund et al | 2017 | Systematic review of social media interventions for smoking cessation | US | Secondary | To determine whether social media interventions for smoking cessation are feasible, acceptable, and potentially effective; to identify approaches for recruiting subjects; and to examine the specific intervention design components and strategies employed to promote user engagement and retention. |
| Tufte and Babic | 2017 | Effect of Social Media in a mHealth Application | Europe | Primary | To review the potential of social media in  how it can promote a healthy lifestyle utilized in an app and to evaluate the app in order to meet  usability requirements. ^b^ |
| Dhar | 2018 | Social Media and Its Implications in Pediatric Dentistry | US | Secondary | To review social media’s implications in pediatric dentistry. ^b^ |
| Gabarron et al | 2018 | Social media for health promotion in diabetes: study protocol for a participatory public health intervention design | Europe | Primary | To research how to best use social media to promote healthy lifestyles with and within the Norwegian population. |
| Leung et al | 2018 | Social Media Users' Perception of Telemedicine and mHealth in China: Exploratory Study | Asia | Primary | To examine a social media platform with a dedicated focus on health information technology and informatics in China and to utilize the findings to support further research. |
| Li et al | 2018 | An analysis of stigma and suicide literacy in responses to suicides broadcast on social media | Asia | Primary | To detect stigma expressions in social media posts through language use patterns and then identify suicide literacy in responses to such broadcast. |
| Parmar et al | 2018 | Connecting With Your Dentist on Facebook: Patients' and Dentists' Attitudes Towards Social Media Usage in Dentistry | UK | Primary | To explore patients' and dentists' attitudes toward social media usage and their current online behaviors in this context and to examine potential challenges and opportunities regarding dentists' adoption of social media practices. |
| Robinson et al | 2018 | The #chatsafe project. Developing guidelines to help young people communicate safely about suicide on social media: A Delphi study | Australia | Primary | To develop a set of evidence informed guidelines to assist young people to communicate about suicide via social media with the input of young people as active participants of the study. |
| Sarkar et al | 2018 | Using Social Media to Target Cancer Prevention in Young Adults: Viewpoint | US | Secondary | To describe the current landscape of nascent research using social media to target cancer prevention efforts in young adults and propose future directions to strengthen the scientific knowledge supporting social media strategies to promote cancer prevention behaviors. |
| van den Heuvel et al | 2018 | eHealth as the Next-Generation Perinatal Care: An Overview of the Literature | Europe | Secondary | To review the current literature on eHealth developments in pregnancy to assess this new generation of perinatal care. |
| Alalawi et al | 2019 | The Effect Of Social Media On The Choice Of Dental Patients: A Cross-Sectional Study In The City Of Jeddah, Saudi Arabia | Middle East | Primary | To investigate the important factors of patients when choosing a dental practice, and the effectiveness of a social media presence for a dental practice to engage with and obtain new patients, as well as the return on investment of social media marketing. |
| Alanzi and Alsaeed | 2019 | Use of Social Media in the Blood Donation Process in Saudi Arabia | Middle East | Primary | To investigate the use of social media in the blood donation process in Saudi Arabia. |
| Coates et al | 2019 | Social Media Influencer Marketing and Children's Food Intake: A Randomized Trial | UK | Primary | To examine the impact of social media influencer marketing of foods on children’s food intake. |
| Curtis et al | 2019 | Comparison of Smartphone Ownership, Social Media Use, and Willingness to Use Digital Interventions Between Generation Z and Millennials in the Treatment of Substance Use: Cross-Sectional Questionnaire Study | US | Primary | To compare digital platforms used among adolescents and emerging adults attending outpatient substance use treatment and to examine receptiveness toward these platforms in order to support substance use treatment and recovery. |
| De Benedictis at al | 2019 | WhatsApp in hospital? An empirical investigation of individual and organizational determinants to use | Europe | Primary | To assess if and how individual and organizational determinants can trigger or inhibit the use of WhatsApp in a hospital setting, and which variables managers can exploit to guide professionals’ behaviors. |
| DeSmet | 2019 | Social media and lifestyles in youth mental health promotion | Europe | Primary | To discuss social media’s effect on youth mental health. ^b^ |
| Dorfman et al | 2019 | Google Ranking of Plastic Surgeons Values Social Media Presence Over Academic Pedigree and Experience | US | Primary | To analyze the respective importance of physician academic pedigree, experience, and social media presence on plastic surgeon Google first-page search result placement. |
| Nayak and  Linkov | 2019 | Social Media Marketing in Facial Plastic Surgery: What Has Worked? | US | Secondary | To review best practices in utilizing social media in marketing for plastic surgery. ^b^ |
| Nayyar et al | 2019 | Are You on the Right Platform? A Conjoint Analysis of Social Media Preferences in Aesthetic Surgery Patients | US | Primary | To evaluate social media preferences of patients seeking aesthetic surgery. |
| Ramo et al | 2019 | Development of a social media-based intervention targeting tobacco use and heavy episodic drinking in young adults | US | Primary | To develop and test usability of the smoking tobacco and drinking intervention for young adults delivered on Facebook. |
| Simplicio | 2019 | Social media and Dentistry: ethical and legal aspects | Latin America | Secondary | To contribute to the discussion and elucidation of questions associated with advertising that uses clinical images and photographs of patients in social media for commercial purposes and self-promotion. |
| Smith and Magnani | 2019 | New technologies, new disparities: The intersection of electronic health and digital health literacy | US | Secondary | to identify challenges with digital health literacy and outline solutions to improve access to digital health services and their use for individuals with limited digital health literacy. |
| Todorovic et al | 2019 | Can social media intervention improve physical activity of medical students? | Europe | Primary | To assess physical activity level among first- and fifth-year medical students and social media intervention with the aim to improve physical activity among them. |
| Viner et al | 2019 | Roles of cyberbullying, sleep, and physical activity in mediating the effects of social media use on mental health and wellbeing among young people in England: a secondary analysis of longitudinal data | UK | Secondary | To explore associations between the frequency of social media use and later mental health and wellbeing in adolescents, and how these effects might be mediated. |
| Abd-Alrazaq et al | 2020 | Top Concerns of Tweeters During the COVID-19 Pandemic: Infoveillance Study | Middle East | Primary | To identify the main topics posted by Twitter users related to the COVID-19 pandemic. |
| Allington et al | 2020 | Health-protective behaviour, social media usage and conspiracy belief during the COVID-19 public health emergency | US | Primary | To determine the relationship between conspiracy belief and health protective behaviour and the relationship between conspiracy belief and social media usage. ^b^ |
| Chan et al | 2020 | Social media for rapid knowledge dissemination: early experience from the COVID-19 pandemic | Asia | Secondary | To describe an example of an efficient and rapidly disseminated infographic describing a practical intubation guideline for use in operating theatres and other critical care areas during a pandemic. ^b^ |
| Cherian et al | 2020 | Content shared on social media for national cancer survivors day 2018 | US | Primary | To explore the discourses surrounding cancer survivorship on social media, paying particular attention to how individuals who identify as cancer survivors represent their experience. |
| Depoux et al | 2020 | The pandemic of social media panic travels faster than the COVID-19 outbreak | Europe | Primary | To discuss social media panic amidst the COVID-19 pandemic. ^b^ |
| Gao et al | 2020 | Mental health problems and social media exposure during COVID-19 outbreak | Asia | Primary | To describes the prevalence and distribution of two major mental disorders-anxiety and depression among Chinese population, and examine their associations with social media exposure by rapid assessment during COVID-19 outbreak. |
| Gonzalez-Padilla and  Tortolero-Blanco | 2020 | Social media influence in the COVID-19 Pandemic | Europe | Secondary | To summarize the most relevant information on the influence, and advantages, and disadvantages of the use of social networks during the COVID-19 pandemic. |
| Puri et al | 2020 | Social media and vaccine hesitancy: new updates for the era of COVID-19 and globalized infectious diseases | Canada | Secondary | To discuss the current position of social media platforms in propagating vaccine hesitancy and explore next steps in how social media may be used to improve health literacy and foster public trust in vaccination. |
| Robinson et al | 2020 | Globalization of the #chatsafe guidelines: Using social media for youth suicide prevention | Australia | Primary | To describe the adaptation of the Australian #chatsafe guidelines for an international audience, and their initial roll out via social media. |
| Stellefson et al | 2020 | Evolving Role of Social Media in Health Promotion: Updated Responsibilities for Health Education Specialists | US | Secondary | To examine the method with which social media users’ access, negotiate, and create health information that is both actionable and impactful for diverse audiences, strategies for overcoming challenges to using social media in health promotion and best practices for designing, implementing, and evaluating social media forums in public health. |
| Wang et al | 2020 | Immediate Psychological Responses and Associated Factors during the Initial Stage of the 2019 Coronavirus Disease (COVID-19) Epidemic among the General Population in China | Asia | Primary | To survey the general public in China to better understand their levels of psychological impact, anxiety, depression, and stress during the initial stage of the COVID-19 outbreak. |
| Wilson and  Wiysonge | 2020 | Social media and vaccine hesitancy | US | Primary | To evaluate the effect of social media and online foreign disinformation campaigns on vaccination rates and attitudes towards vaccine safety. |
| Farsi | 2021 | Social Media and Health Care, Part I: Literature Review of Social Media Use by Health Care Providers | Middle East | Secondary | To shed light on social media use worldwide and to discuss how it has been used as an essential tool in the health care industry from the perspective of health care providers. |

^a^ Primary evidence includes randomized control studies, cohort studies, case control studies and case reports. Secondary evidence includes meta-analysis, systematic reviews, narrative reviews, coping review, short communications, commentaries, viewpoint papers and overviews

^b^ objective/s was not stated clearly in the publication and was concluded by the authors.
